# Using kinematic analyses to explore sensorimotor control impairments in children with 22q11.2 deletion syndrome

**DOI:** 10.1186/s11689-019-9271-3

**Published:** 2019-06-10

**Authors:** Adam C. Cunningham, Liam Hill, Mark Mon-Williams, Kathryn J. Peall, David E. J. Linden, Jeremy Hall, Michael J. Owen, Marianne B. M. van den Bree

**Affiliations:** 1 MRC Centre for Neuropsychiatric Genetics and Genomics, Division of Psychological Medicine and Clinical Neurosciences, Cardiff University School of Medicine, Haydn Ellis Building, Maindy Road, Cathays, Cardiff, CF24 4HQ UK; 2 School of Psychology, University of Leeds, Leeds, West Yorkshire UK

**Keywords:** 22q11.2 deletion syndrome, Movement difficulties, Coordination, ADHD, ASD, Anxiety

## Abstract

**Background:**

The 22q11.2 deletion is associated with psychiatric and behavioural disorders, intellectual disability and multiple physical abnormalities. Recent research also indicates impaired coordination skills may be part of the clinical phenotype. This study aimed to characterise sensorimotor control abilities in children with 22q11.2 deletion syndrome (22q11.2DS) and investigate their relationships with co-occurring IQ impairments and psychopathology.

**Methods:**

Fifty-four children with 22q11.2DS and 24 unaffected sibling controls, comparable in age and gender, underwent kinematic analysis of their hand movements, whilst performing a battery of three visuo-manual coordination tasks that measured their tracking, aiming and steering abilities. Additionally, standardised assessments of full-scale IQ (FSIQ), attention deficit hyperactivity disorder, indicative autism spectrum disorder (ASD) and anxiety disorder symptomatology were conducted.

**Results:**

Children with 22q11.2DS showed deficits on seven of eight kinematic descriptors of movement quality across the three coordination tasks, compared to controls. Within 22q11.2DS cases, the extent of impairment on only three kinematic descriptors was significantly related to FSIQ after correction for multiple testing. Moreover, only error whilst visuo-manually tracking was nominally associated with ADHD symptom counts.

**Conclusions:**

Impairments in sensorimotor control are seen on a range of visuo-manual tasks in children with 22q11.2DS but the extent of these impairments are largely unrelated to the severity of other psychopathological and intellectual impairments commonly found in children with 22q11.2DS.

**Electronic supplementary material:**

The online version of this article (10.1186/s11689-019-9271-3) contains supplementary material, which is available to authorized users.

## Background

22q11.2 deletion syndrome (22q11.2DS) is a chromosomal microdeletion disorder caused by a hemizygous microdeletion on the long arm of chromosome 22. It affects 1 in 2000–4000 live births, though the rate in low-risk pregnancies is as high as 1 in 992 [1]. The deletion is associated with developmental delay and increased risk of physical abnormalities and mental disorders [2]. Impaired motor skills are gaining recognition as a feature of 22q11.2DS too, after one of the largest studies to date to assess coordination skills in children with 22q11.2DS [3] found they scored significantly lower than sibling controls on a parental-report screening questionnaire, with 81% surpassing the threshold score for suspected developmental coordination disorder (DCD), compared to 6% of controls. DCD is a neurodevelopmental disorder characterised by specific difficulties with learning and performing coordinated movements, not otherwise explained by co-occurring physical or neurological impairments [4]. An increased risk of coordination skill deficits in children with 22q11.2DS is consistent with earlier evidence indicating they typically achieve gross-motor milestones later [5], have abnormal development in areas of the brain associated with sensorimotor control [6, 7] and demonstrate highly variable profiles on motor skill assessment batteries (such as the Movement ABC-2), although without specific sub-domains emerging as being consistently affected [3, 8].

Mild to moderate intellectual difficulties are common in children with 22q11.2DS, and low IQ is associated with performance on motor assessments in 22q11.2DS [3, 9]. However, the degree to which IQ impairments account for poor performance remains unclear due to conflicting reports of significant motor impairments persisting in 22q11.2DS even in comparisons with IQ-matched controls [10, 11]. (Although children with 22q11.2DS out-perform IQ-matched controls on some visuo-manual tasks [12]). Similarly, 22q11.2DS is a well-established risk factor for several other neurodevelopmental disorders [13] that have the potential to confound performance on motor skill assessments, e.g. ADHD and ASD [14]. Thus, the extent to which coordination difficulties in children with 22q11.2DS are attributable primarily to other non-sensorimotor forms of co-occurring psychopathology remains unclear.

Given the complex picture of co-occurring deficits in 22q11.2DS, more objective and specific assessments of fundamental sensorimotor processing abilities are needed to determine as precisely as possible the sensorimotor difficulties that are present in the syndrome. However, only one study to date (reported across two papers [15, 16] has used kinematic analysis techniques to describe movement quality in 21 children with 22q11.2DS. This revealed comparatively greater temporal and spatial errors compared to IQ-matched controls on a rhythmic visuo-manual tracking task, with the 22q11.2DS group’s kinematics suggesting the use of a more developmentally immature ‘ballistic’ movement strategy. Consequently, claims that sensorimotor control processes are directly affected in 22q11.2DS could be further strengthened by determining whether differences are consistently observable in the kinematics of children with 22q11.2DS whilst performing basic coordination tasks, which have limited cognitive demands.

The present study conducted a more extensive and detailed assessment of the underlying sensorimotor control abilities of children with 22q11.2DS and unaffected sibling controls than previously attempted; utilising a battery of computerised tasks that assessed several fundamental coordination behaviours (aiming, tracking and steering) whilst recording precise end-point kinematic response data. Data on global development were also obtained, enabling us to test whether children with 22q11.2DS exhibited consistent evidence of compromised sensorimotor control abilities; and the extent to which any such impairments were related to IQ level and/or the co-occurring psychiatric symptoms common within children with 22q11.2DS [17].

## Methods

### Participants and procedure

Fifty-four participants with 22q11.2DS (mean age 13.73 years; age range 6.45–18.56 years) and 24 unaffected siblings (mean 12.99 years; range 8.50–16.95 years) who were comparable in age and gender (see Table 1) were recruited via UK Medical Genetics clinics, word of mouth and advertisements through 22q11.2DS charities, using recruitment protocols approved by the NHS Ethics and Research and Development committees. Informed consent was obtained prior to recruitment from the carers of the children. Inclusion criteria were age of 6 years or older (in order for psychiatric assessments to be valid) and confirmed the presence of the 22q11.2 deletion in the child with the deletion. Presence of the 22q11.2 deletion between either low copy repeat regions A and B or A and D was confirmed by medical genetics laboratories or by the Cardiff University MRC Centre for Neuropsychiatric Genetics and Genomics using microarray techniques. All participants completed a battery of sensorimotor tasks, along with a standardised assessment of IQ, and assessments of attention deficit hyperactivity disorder (ADHD), indicative autism spectrum disorder (ASD) and anxiety symptoms (see the “Assessments” section for details). All assessments were completed either in the participants’ homes or at the laboratory at Cardiff University.

**Table 1:** Demographic information and summary statistics for age, IQ and psychopathology symptoms

	22q11.2DS			Controls				
	n	Mean (range)	SD	*n*	Mean (range)	SD	*t*	*p*
Age	54	13.73 (6.45-18.56)	3.44	24	12.99 (8.50–16.95)	2.52	1.06	.292
FSIQ	54	72.06 (51–105)	13.13	24	108.25 (63–139)	18.16	− 8.79	< .001
	n	Median	IQR	n	Median	IQR	W	*p*
ADHD symptoms	54	3	7.75	24	0	0.00	1761	< .001
Indicative ASD symptoms	53	9	9.00	23	1	3.00	2545	< .001
anxiety symptoms	54	1	6.00	24	0	2.00	1198	.039
Mother’s ethnic background	n (families)	%						
Caucasian	54	93.1						
Other	4	6.9						
Mother’s education level	n	%						
High (University degree and/or other postgraduate qualification)	12	20.7						
Low (O-levels, GCSE’s)	10	17.2						
Middle (A-level’s, highers, vocational training)	29	50.0						
No school leaving exams	4	6.9						
Unknown	3	5.2						
Approximate family income	*n* (families)	%						
<=£19,999	8	13.8						
£20,000–£39,999	14	24.1						
£40,000–£59,999	16	27.6						
£60,000+	14	24.1						
Unknown	6	10.3						

Note: 148 families took part, and 4 families provided only a sibling control

*FSIQ* full-scale IQ, *ADHD* attention deficit hyperactivity disorder, *ASD* autism spectrum disorder, *22q11.2DS* 22q11.2 deletion syndrome

### Assessments

#### Sensorimotor control

Each participant completed the Clinical Kinematic Assessment Tool (CKAT) [18], a standardised computerised battery comprising three sub-tests of visuo-manual sensorimotor control. All sub-tests required participants use a handheld stylus to interact with 2D visual stimuli presented on a tablet computer. Outcome measures for each sub-test were as follows.

*Tracking* required participants to keep their stylus as close as possible to the centre of a circular target (5 mm diameter) as it moved in a sinusoidal figure-8 pattern, at three increasing speeds, for 3 min, under two conditions: one with a guide path illustrating the target’s trajectory, another without this additional assistance (presented first). Performance was described by tracking error (TE): the straight-line distance in millimetres from the moving target’s centre-point to the tip of the stylus, sampled at a rate of 120 Hz for the task’s duration. For analysis, TE was summarised by the mean and standard deviation (termed intra-individual variability (IIV) hereafter) of this time series of response.

*Aiming* required participants to respond as quickly and accurately as possible to fifty consecutively displayed 5 mm diameter circular ‘targets’ appearing on-screen. Both preparatory and online components of response within each discrete aiming movement were captured, via measurements of: reaction time (RT) and time to peak speed (TPS) in seconds, peak speed (PS) in millimetres per second, and normalised jerk index (NJ), a measure of “smoothness” of the movement profile [19]. Participant’s median responses on each of these outcomes were analysed.

*Steering* assessed the ability to exert precise force control to produce complex multi-component movements of the stylus under time-constraints. Participants were instructed to move their stylus along a 4-mm-wide path (comprising an angular combination of straight-line and curved trajectories) from an on-screen ‘start’ to ‘finish’ zone, whilst minimising deviation from the path and also trying to stay within a transparent ‘pacing’ box. The ‘pacing’ box highlighted a smaller portion of the overall path and moved along it at a fixed speed from the start to finish, taking 36 s to do so. Path accuracy (PA) was measured as the mean error in millimetres between stylus position and the centre of the idealised reference path at each sampled point (at 120 Hz). Completion time (CT) was the time taken to reach the finish zone. Median value across six trials for each of these outcomes was analysed.

For supplementary details regarding CKAT battery tasks and their kinematic outcomes see establishing papers: Flatters et al. [18] and Culmer et al. [19].

#### Psychometric and psychopathological assessment

Full-scale IQ (FSIQ) was obtained by administering the Wechsler Abbreviated Scale of Intelligence (four subtests) (WASI) [20]. Parents completed the Social Communication Questionnaire (SCQ) [21], with responses summed into a continuous score (0–39) of symptomatology that is indicative of ASD. An individual was classed as having indicative ASD if they scored 15 or more on the SCQ. Additionally, the research-diagnostic Child and Adolescent Psychiatric Assessment (CAPA) [22] interview was conducted with the primary caregiver to measure anxiety and attention-deficit/hyperactivity disorder symptoms and assign a DSM-5 research diagnosis of these disorders. Symptoms were counted as present if an individual scored ≥ 2 on the relevant question. Anxiety symptoms included any symptom of generalised anxiety disorder, social phobia, specific phobia, separation anxiety, panic disorder with and without agoraphobia, agoraphobia, and obsessive-compulsive disorder.

## Statistical analysis

Before statistical analysis, all raw scores for kinematic (i.e. CKAT) outcome variables except peak speed (PS) and completion time (CT) were reciprocally transformed to resolve outliers and normalise the distributions. Statistical analysis was carried out in R-3.5.1 [23] on OSX-10.14.2.

*Analysis 1* investigated differences in sensorimotor control between groups (effect of deletion status) for each kinematic outcome variable after specifying age and gender as covariates, due to their well-established effects on sensorimotor performance [24]. We did not include FSIQ as a covariate in these analyses as it is strongly correlated with deletion carrier status. For outcomes relating to tracking, these ANCOVAs also examined whether deletion status interacted with other manipulations of task difficulty (i.e. variations in target-speed and presence of a guide path). *Analysis 2* used hierarchical linear regressions to investigate, within the children with 22q11.2DS specifically, the contribution of FSIQ to explaining performance on those kinematic outcomes where an effect of deletion status had been found in Analysis 1. These models controlled for age and gender at step one, then added FSIQ at a second step. *Analysis 3* utilised equivalent hierarchical models, this time investigating at the second step the relationship between kinematic outcomes and symptom counts for ADHD, ASD, and anxiety disorders in the 22q11.2DS group.

A Bonferroni-Holm correction was used to correct *p* values for the number of comparisons made, and adjusted *p* values are reported alongside original *p* values in the text and tables.

## Results

See Table 1 for summary demographic information. One child with 22q11.2DS was receiving Aripiprazole for psychosis and two children with 22q11.2DS were receiving methylphenidate. No other relevant medication use was noted. Indicative ASD symptoms were not available for one child with 22q11.2DS and one unaffected sibling control. One child with 22q11.2DS had the smaller (1.5 Mb) A-B deletion, whilst the remaining children had the typical (3 Mb) A-D deletion.

### Analysis 1: sensorimotor control in 22q11.2DS and sibling controls

#### Tracking

**Fig. 1:**
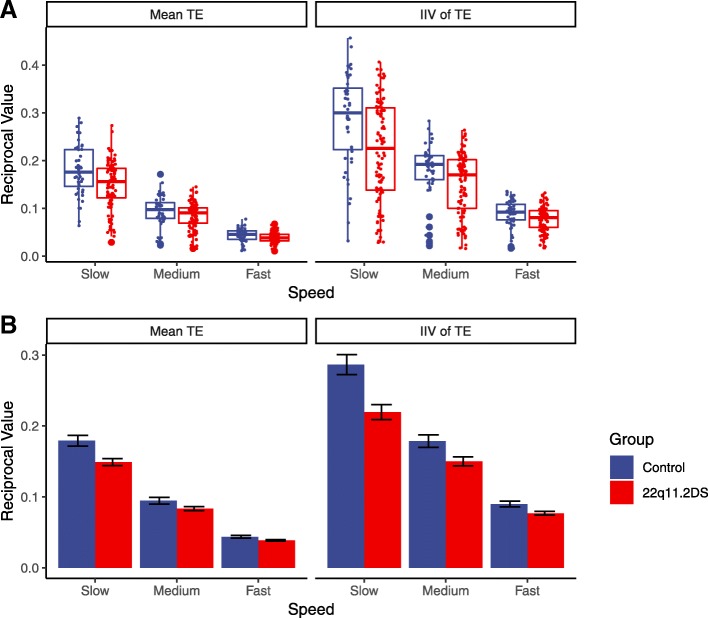
**a** Boxplots of reciprocal mean tracking error (TE) and reciprocal intra-individual variability of tracking error, by group and speed. **b** Reciprocal mean tracking error (mean TE) and intra-individual variability of TE (IIV of TE) by group and speed. Notes: error bars indicate standard error of the mean; reciprocal transforms mean larger quantities in these graphs denote less error

ANCOVA results indicated that visuo-manual tracking performance, in terms of both its average and intra-individual variability (i.e. mean tracking error and standard deviation of tracking error), was poorer in children with 22q11.2DS compared to siblings, with small to moderate effect sizes (see Table 2 and Fig. 1a). Interaction terms within these models also indicated a significant interaction between deletion status and target speed for both mean (*F* = 6.80, df = 2, *p =* .001, η_p_^2^ = .029) and standard deviation (*F* = 7.08, df = 1, *p =* .001, η_p_^2^ = .030) of tracking error. All other interactions between deletion status and experiment manipulations on this sub-test were not significant (*p* > .0056). These interactions arose due to the deficits in the performance between carriers and siblings reducing as target speed increased. This is demonstrated in Fig. 1b.

**Table 2:** Mean performance on kinematic outcomes for children with 22q11.2 deletion (*n* = 54) and controls (*n* = 24)

		22q11.2DS		Controls						
Task	Outcome	Mean	SD	Mean	SD	df	*F*	*p*	*p*_adj_	η_p_^2^
Tracking	Mean error	0.089	0.056	0.106	0.067	1	40.812	< .001	< .001	.082
	IIV of error	0.147	0.094	0.185	0.105	1	46.617	< .001	< .001	.093
Aiming	Peak speed	324.286	75.247	379.144	106.222	1	7.756	.007	.028	0.095
	Time to peak speed	1.7	0.267	1.845	0.296	1	10.482	.002	.012	.124
	Normalised jerk	0.004	0.001	0.005	0.002	1	27.655	< .001	< .001	.272
	Reaction time	2.848	0.442	3.03	0.477	1	9.846	.002	.013	.117
Steering	Path accuracy	0.76	0.203	0.916	0.196	1	19.132	< .001	< .001	.205
	Completion time	36.057	6.357	36.317	4.396	1	0.152	.698	.770	.002

*F* values and statistics indicate main effect of deletion status for the models reported in the “Analysis 1: sensorimotor control in 22q11.2DS and sibling controls” section of this paper

*p*_adj_ indicates p value after Bonferroni-Holm adjustment

Effect size thresholds: η_p_^2^ > .01 small, > .09 medium, > .25 large [25]

22q11.2DS: 22q11.2 deletion syndrome; IIV = intra-individual variability

#### Aiming

ANCOVA results also illustrated that 22q11.2 deletion carriers’ aiming movements comprised a longer preparatory phase (longer reaction times) and, once initiated, exhibited increased normalised jerk and time to peak speed compared to controls, with medium-to-large effect sizes (Table 2). Deletion carriers had a lower peak speed compared to controls, with a medium effect size.

#### Steering

ANCOVAs of steering performance revealed no difference between deletion carriers and siblings for completion time but there were significant moderately sized differences in path accuracy, with children with the deletion, on average, further away from the ideal path (Table 2).

### Analysis 2: sensorimotor control and IQ in children with 22q11.2DS

Hierarchical regression models were constructed for each of the six kinematic outcome measures where a significant effect of deletion status was found in Analysis 1. These outcomes were first regressed on age and gender, before the addition of FSIQ. For mean tracking error and its intra-individual variability, average performance across both conditions at the slowest target speed was analysed due to the interactions with speed observed in Analysis 1.

These models showed that addition of FSIQ increased predictive power for mean tracking error (*R*^2^ change = .09, *F* = 7.15, df = 1, *p =* .010, *p*_adj_ = .040), time to peak speed (*R*^2^ change = 0.10, *F* = 8.43, df = 1, *p =* .005, *p*_adj_ *=* .025) and path accuracy (*R*^2^ change = 0.12, *F* = 10.69, df = 1, *p =* .002 *p*_adj_ = .012), after corrections for multiple comparisons were applied. Before correction, nominal associations between FSIQ and intra-individual variability of tracking error (*R*^2^ change = .09, *F* = 6.00, df = 1, *p =* .018, *p*_adj_ = .064) and peak speed (*R*^2^ change = .10, *F* = 5.29, df = 1, *p =* .026, *p*_adj_ = .077) were observed. No other relationships were found. Full results of these regression analyses are presented in Additional file 1: Table S1.

### Analysis 3: sensorimotor control and co-occurring psychopathology with 22q11.2DS

Hierarchical models of each kinematic outcome regressed on the number of ADHD, indicative ASD or anxiety symptoms observed, after controlling for age and gender, revealed nominal (*p* < .05) associations between increased ADHD symptoms (*R*^2^ change = .07, *F* = 5.31, df = 1, *p =* .025, *p*_adj_ *=* .077) and increased mean tracking error. All other relationships were non-significant before and after correction for multiple comparisons. Full results of these regression analyses are presented in Additional file 2: Table S2.

Sensitivity analyses, excluding the three individuals receiving medication or the one carrying the 1.5Mb deletion from analyses 2 and 3 identified an additional nominal (*p* < .05) association between anxiety symptoms and path accuracy in children carrying the 22q11.2 deletion (*R*^2^ change = .06, *F* = 4.66, df = 1, *p =* .036, *p*_adj_ *=* .093). All other results remained the same.

## Discussion

The current study presents the most extensive and detailed investigation of sensorimotor control abilities in children with 22q11.2DS to date, in a sample more than twice the size of the only previous study to use objective kinematic analysis techniques [15, 16]. Movement kinematics for tracking, aiming and steering all indicated significant differences in these basic sensorimotor control behaviours in children with 22q11.2DS compared to their siblings.

Rhythmic visuo-manual tracking deficits were expected given earlier work [16], but these deficits have been explored in more detail, allowing group differences in average accuracy and intra-individual variability to be established. Deficits in intra-individual variability were of equal, if not greater, size to between-group differences in average accuracy. This finding is interesting given increasing intra-individual variability is a noted cognitive symptom of several degenerative movement disorders [26, 27], along with ADHD [25] and might indicate difficulties in maintaining stable performance levels within tasks [28, 29]. Related deficits in accuracy (but not speed) whilst steering, and smoothness of movement whilst aiming, also suggest that sensorimotor deficits in children with 22q11.2DS reflect specific problems with integrating feedback in a timely manner in order to make fluent online corrections. Abnormal jerk profiles have also been shown repeatedly to arise in individuals with degenerative movement disorders such as Parkinson’s disease [30, 31]. Such kinematic discrepancies are also consistent with other research finding coordination deficits relative to controls in children with 22q11.2DS lessen on tasks where time to respond is unconstrained [12]. These patterns of results may suggest that there is a disruption in the ability to build and refine internal models for guiding and supervising the action.

The small number of significant relationships between kinematic variables and FSIQ and the limited variance in sensorimotor ability they explain (10–12% when significant) suggests that co-occurring IQ deficits are not the sole contributor to coordination difficulties in 22q11.2DS. Only accuracy when steering, time to peak speed when performing aimed movements and error when tracking an object were found to be related to FSIQ. If IQ impairment was causative of sensorimotor deficits in 22q11.2DS, we would expect the severity of sensorimotor deficits to increase with the level of IQ impairment across many measures. The evidence presented here would suggest that in this population, where mild or moderate intellectual difficulties are common, motor difficulties are at most weakly associated with IQ level.

Lastly, we found only nominal relationships between ADHD symptoms and average tracking error. The absence of consistent significant relationships between sensorimotor performance on the one hand and psychopathology or IQ on the other suggests that sensorimotor impairment is not a consequence of generally poorer functional or coping levels but a specific component of the phenotype of 22q11.2DS. This result differs from previous research, where psychopathology symptoms were found to be strongly related to overall coordination [3]; however, this earlier study relied on parental report, whilst the current study used objective and direct methods to assess sensorimotor control. In addition, the questionnaire used in the previous study, the developmental coordination disorder questionnaire (DCDQ) probes about more general aspects of movement including problems that affect daily life, such as writing or ball skills. Some of these aspects might be more likely to be affected by psychopathology, such as being nervous about feeling clumsy. Therefore, whilst it is reasonable to suspect that sensorimotor problems may underlie poor scores on the DCDQ, it is also likely that they would not explain the full scope of scores. Resolving this difference is important for the clinical evaluation of motor problems in 22q11.2DS.

It should be noted that 22q11.2DS is a complex disorder, with many associated physical health conditions [2], and unfortunately, it was not possible to assess all neurological and/or musculoskeletal problems that might contribute to the sensorimotor deficits demonstrated here. However, in light of the present findings and the increasing evidence for movement disorders in 22q11.2DS across the lifespan [32], we suggest it is appropriate that formal motor assessments are added to the research and clinical standards for 22q11.2DS. This should facilitate earlier detection of movement disorders and implementation of appropriate help. This is important as movement difficulties in childhood have been shown to be related to greater problems in adulthood in non-genotyped populations [33, 34]. However, it is currently not known how early difficulties with motor skills can be identified in this population. The difficulty in reliably identifying coordination impairments in very young children (under 5 years old) is even commented on in the most recent clinical recommendations for diagnosing DCD [4]. Indeed, which assessments would be the most sensitive and useful for this population are important research questions that should be addressed in the future.

## Conclusions

In the present study, we have demonstrated a series of atypical sensorimotor control behaviours in children with 22q11.2DS that provide a plausible explanation for the deficits in coordination that have been noted in the syndrome. In addition, we highlight the relative independence of sensorimotor deficits from co-occurring IQ deficits and other potential associations with specific domains of psychopathology. This work further demonstrates the importance of sensorimotor difficulties in children with 22q11.2DS and strongly suggests that neurodevelopmental disorders of movement should be considered part of the clinical phenotype in 22q11.2DS.

## Additional files

Additional file 1: **Table S1.** Hierarchical regression results for Analysis 2, where sensorimotor outcome measures are predicted by full-scale IQ, with age and sex as covariates. (DOCX 18 kb)

Additional file 2: **Table S2.** Hierarchical regression results for Analysis 3, where sensorimotor outcome measures were predicted by ADHD symptoms, indicative ASD symptoms and anxiety symptoms, with age and gender as covariates (DOCX 51 kb)

## Data Availability

The datasets used and/or analysed during the current study are available from the corresponding author on reasonable request.

## Abbreviations

22q11.2DS: 22q11.2 deletion syndrome
ADHD: Attention deficit hyperactivity disorder
ASD: Autism spectrum disorder
CAPA: Child and Adolescent Psychiatric Assessment
CKAT: Clinical Kinematic Assessment Tool
DCD: Developmental coordination disorder
FSIQ: Full-scale IQ
IIV: Intra-individual variability
SCQ: Social Communication Questionnaire
WASI: Weschler Abbreviated Scale of Intelligence
