# Supplementary material for: Using kinematic analyses to explore sensorimotor control impairments in children with 22q11.2 deletion syndrome
Source: J Neurodev Disord. 2019 Jun 10;11:8. doi: 10.1186/s11689-019-9271-3 (PMC6558818; doi:10.1186/s11689-019-9271-3)
Supplement: Supplementary file 1 — Table S1. Hierarchical regression results for Analysis 2, where sensorimotor outcome measures are predicted by full-scale IQ, with age and sex as covariates. (DOCX 18 kb) [file 11689_2019_9271_MOESM1_ESM.docx]

Additional file 1: Table S1. Hierarchical regression results for analysis 2, where sensorimotor outcome measures are predicted by Full scale IQ, with age and sex as covariates.

| **TPS** | **Model 1** | | | | **Model 2** | | | |
| --- | --- | --- | --- | --- | --- | --- | --- | --- |
|  | B | SE | Std.B | p | B | SE | Std.B | p |
| Constant | 1 | 0.14 |  | 1.43e-09 | 0.5 | 0.23 |  | 0.0339 |
| Age | 0.044 | 0.0092 | 0.57 | 1.45e-05 | 0.049 | 0.0088 | 0.63 | 9.11e-07 |
| Sex | 0.087 | 0.063 | 0.16 | 0.175 | 0.097 | 0.059 | 0.18 | 0.106 |
| FSIQ |  | | | | 0.0065 | 0.0022 | 0.32 | 0.00549 |
| R2 | 0.31 |  | | | 0.41 |  | | |
|  | R2 Change | F | p | adj.p |  | | | |
|  | 0.099 | 8.4 | 0.005 | 0.025 |  |  |  |  |
| **RT** | **Model 1** | | | | **Model 2** | | | |
|  | B | SE | Std.B | p | B | SE | Std.B | p |
| Constant | 1.5 | 0.21 |  | 1.45e-09 | 0.96 | 0.35 |  | 0.00805 |
| Age | 0.09 | 0.013 | 0.7 | 1.56e-08 | 0.095 | 0.013 | 0.74 | 3.52e-09 |
| Sex | 0.17 | 0.092 | 0.2 | 0.0665 | 0.18 | 0.09 | 0.21 | 0.0462 |
| FSIQ |  | | | | 0.0067 | 0.0034 | 0.2 | 0.0544 |
| R2 | 0.47 |  | | | 0.51 |  | | |
|  | R2 Change | F | p | adj.p |  | | | |
|  | 0.038 | 3.9 | 0.054 | 0.142 |  |  |  |  |
| **PA** | **Model 1** | | | | **Model 2** | | | |
|  | B | SE | Std.B | p | B | SE | Std.B | p |
| Constant | 0.35 | 0.11 |  | 0.0018 | -0.1 | 0.17 |  | 0.555 |
| Age | 0.032 | 0.0069 | 0.54 | 2.97e-05 | 0.036 | 0.0065 | 0.61 | 1.12e-06 |
| Sex | -0.048 | 0.047 | -0.12 | 0.315 | -0.039 | 0.044 | -0.097 | 0.37 |
| FSIQ |  | | | | 0.0054 | 0.0017 | 0.35 | 0.00196 |
| R2 | 0.33 |  | | | 0.45 |  | | |
|  | R2 Change | F | p | adj.p |  | | | |
|  | 0.12 | 11 | 0.002 | 0.012 |  |  |  |  |

TE: Tracking Error, IIV of TE: Intra-Individual Variability of Tracking Error, NJ: Normalised Jerk, TPS: Time to Peak Speed, RT: Reaction Time, PA: Path Accuracy
