# Supplementary material for: Using kinematic analyses to explore sensorimotor control impairments in children with 22q11.2 deletion syndrome
Source: J Neurodev Disord. 2019 Jun 10;11:8. doi: 10.1186/s11689-019-9271-3 (PMC6558818; doi:10.1186/s11689-019-9271-3)
Supplement: Supplementary file 2 — Table S2. Hierarchical regression results for Analysis 3, where sensorimotor outcome measures were predicted by ADHD symptoms, indicative ASD symptoms and anxiety symptoms, with age and gender as covariates (DOCX 51 kb) [file 11689_2019_9271_MOESM2_ESM.docx]

Additional file 2: Table S2. Hierarchical regression results for analysis 3, where sensorimotor outcome measures were predicted by ADHD symptoms, indicative ASD symptoms and anxiety symptoms, with age and gender as covariates

| **Mean TE** |  | **Model 1** |  |  |  | **Model 2** |  |  |
| --- | --- | --- | --- | --- | --- | --- | --- | --- |
|  | B | SE | Std.B | p | B | SE | Std.B | p |
| Constant | 0.053 | 0.026 |  | 0.0446 | 0.084 | 0.028 |  | 0.00441 |
| Age | 0.007 | 0.0017 | 0.51 | 0.000124 | 0.0055 | 0.0017 | 0.4 | 0.00277 |
| Sex | -0.005 | 0.012 | -0.052 | 0.67 | -0.0014 | 0.011 | -0.015 | 0.901 |
| ADHD Count |  |  |  |  | -0.0029 | 0.0013 | -0.29 | 0.0254 |
| R2 | 0.27 |  |  |  | 0.34 |  |  |  |
|  | R2 Change | F | p | adj. p |  |  |  |  |
|  | 0.07 | 5.3 | 0.025 | 0.077 |  |  |  |  |
| **IIV of TE** |  | **Model 1** |  |  |  | **Model 2** |  |  |
|  | B | SE | Std.B | p | B | SE | Std.B | p |
| Constant | 0.044 | 0.056 |  | 0.44 | 0.093 | 0.063 |  | 0.142 |
| Age | 0.013 | 0.0036 | 0.44 | 0.00109 | 0.01 | 0.0039 | 0.36 | 0.0111 |
| Sex | -0.0055 | 0.025 | -0.028 | 0.827 | 0.00024 | 0.025 | 0.0012 | 0.992 |
| ADHD Count |  |  |  |  | -0.0047 | 0.0028 | -0.23 | 0.1 |
| R2 | 0.2 |  |  |  | 0.24 |  |  |  |
|  | R2 Change | F | p | adj. p |  |  |  |  |
|  | 0.042 | 2.8 | 0.100 | 0.226 |  |  |  |  |
| **NJ** |  | **Model 1** |  |  |  | **Model 2** |  |  |
|  | B | SE | Std.B | p | B | SE | Std.B | p |
| Constant | 0.00027 | 0.00054 |  | 0.617 | 0.00044 | 0.00062 |  | 0.482 |
| Age | 0.00028 | 3.5e-05 | 0.75 | 1.94e-10 | 0.00027 | 3.8e-05 | 0.72 | 4.47e-09 |
| Sex | -8.7e-05 | 0.00024 | -0.034 | 0.719 | -6.8e-05 | 0.00024 | -0.027 | 0.782 |
| ADHD Count |  |  |  |  | -1.6e-05 | 2.8e-05 | -0.058 | 0.576 |
| R2 | 0.57 |  |  |  | 0.57 |  |  |  |
|  | R2 Change | F | p | adj. p |  |  |  |  |
|  | 0.0027 | 0.32 | 0.576 | 0.740 |  |  |  |  |
| **PS** |  | **Model 1** |  |  |  | **Model 2** |  |  |
|  | B | SE | Std.B | p | B | SE | Std.B | p |
| Constant | 310 | 48 |  | 3.51e-08 | 310 | 55 |  | 1.06e-06 |
| Age | 0.62 | 3.1 | 0.028 | 0.845 | 0.87 | 3.4 | 0.04 | 0.799 |
| Sex | 6.1 | 21 | 0.04 | 0.779 | 5.5 | 22 | 0.036 | 0.804 |
| ADHD Count |  |  |  |  | 0.5 | 2.5 | 0.031 | 0.842 |
| R2 | 0.0019 |  |  |  | 0.0027 |  |  |  |
|  | R2 Change | F | p | adj. p |  |  |  |  |
|  | 8e-04 | 0.04 | 0.842 | 0.866 |  |  |  |  |

| **TPS** |  | **Model 1** |  |  |  | **Model 2** |  |  |
| --- | --- | --- | --- | --- | --- | --- | --- | --- |
|  | B | SE | Std.B | p | B | SE | Std.B | p |
| Constant | 1 | 0.14 |  | 1.43e-09 | 1 | 0.16 |  | 1.06e-07 |
| Age | 0.044 | 0.0092 | 0.57 | 1.45e-05 | 0.046 | 0.01 | 0.59 | 2.98e-05 |
| Sex | 0.087 | 0.063 | 0.16 | 0.175 | 0.083 | 0.064 | 0.16 | 0.204 |
| ADHD Count |  |  |  |  | 0.0035 | 0.0073 | 0.062 | 0.633 |
| R2 | 0.31 |  |  |  | 0.32 |  |  |  |
|  | R2 Change | F | p |  |  |  |  |  |
|  | 0.0032 | 0.23 | 0.633 | 0.760 |  |  |  |  |
| **RT** |  | **Model 1** |  |  |  | **Model 2** |  |  |
|  | B | SE | Std.B | p | B | SE | Std.B | p |
| Constant | 1.5 | 0.21 |  | 1.45e-09 | 1.5 | 0.24 |  | 4.35e-08 |
| Age | 0.09 | 0.013 | 0.7 | 1.56e-08 | 0.09 | 0.015 | 0.7 | 1.37e-07 |
| Sex | 0.17 | 0.092 | 0.2 | 0.0665 | 0.17 | 0.094 | 0.2 | 0.0702 |
| ADHD Count |  |  |  |  | -0.00082 | 0.011 | -0.0087 | 0.939 |
| R2 | 0.47 |  |  |  | 0.47 |  |  |  |
|  | R2 Change | F | p | adj. p |  |  |  |  |
|  | 6.2e-05 | 0.0059 | 0.939 | 0.939 |  |  |  |  |
| **PA** |  | **Model 1** |  |  |  | **Model 2** |  |  |
|  | B | SE | Std.B | p | B | SE | Std.B | p |
| Constant | 0.35 | 0.11 |  | 0.0018 | 0.37 | 0.12 |  | 0.00362 |
| Age | 0.032 | 0.0069 | 0.54 | 2.97e-05 | 0.031 | 0.0075 | 0.52 | 0.000165 |
| Sex | -0.048 | 0.047 | -0.12 | 0.315 | -0.046 | 0.048 | -0.11 | 0.35 |
| ADHD Count |  |  |  |  | -0.0021 | 0.0055 | -0.048 | 0.706 |
| R2 | 0.33 |  |  |  | 0.33 |  |  |  |
|  | R2 Change | F | p | adj. p |  |  |  |  |
|  | 0.0019 | 0.14 | 0.706 | 0.771 |  |  |  |  |

| **Mean TE** |  | **Model 1** |  |  |  | **Model 2** |  |  |
| --- | --- | --- | --- | --- | --- | --- | --- | --- |
|  | B | SE | Std.B | p | B | SE | Std.B | p |
| Constant | 0.045 | 0.026 |  | 0.0931 | 0.054 | 0.029 |  | 0.0647 |
| Age | 0.0074 | 0.0017 | 0.54 | 6.17e-05 | 0.0072 | 0.0017 | 0.52 | 0.000111 |
| Sex | -0.0019 | 0.012 | -0.02 | 0.873 | -0.00014 | 0.012 | -0.0015 | 0.991 |
| Ind. ASD |  |  |  |  | -0.00068 | 0.00083 | -0.1 | 0.419 |
| R2 | 0.29 |  |  |  | 0.3 |  |  |  |
|  | R2 Change | F | p | adj. p |  |  |  |  |
|  | 0.0095 | 0.67 | 0.419 | 0.628 |  |  |  |  |
| **IIV of TE** |  | **Model 1** |  |  |  | **Model 2** |  |  |
|  | B | SE | Std.B | p | B | SE | Std.B | p |
| Constant | 0.025 | 0.057 |  | 0.66 | 0.049 | 0.062 |  | 0.437 |
| Age | 0.014 | 0.0037 | 0.47 | 0.000544 | 0.013 | 0.0037 | 0.46 | 0.000937 |
| Sex | 0.0014 | 0.025 | 0.0071 | 0.956 | 0.0057 | 0.026 | 0.029 | 0.823 |
| Ind. ASD |  |  |  |  | -0.0017 | 0.0018 | -0.12 | 0.347 |
| R2 | 0.22 |  |  |  | 0.24 |  |  |  |
|  | R2 Change | F | p | adj. p |  |  |  |  |
|  | 0.014 | 0.9 | 0.347 | 0.568 |  |  |  |  |
| **NJ** |  | **Model 1** |  |  |  | **Model 2** |  |  |
|  | B | SE | Std.B | p | B | SE | Std.B | p |
| Constant | -1.2e-05 | 0.00053 |  | 0.982 | 0.00036 | 0.00057 |  | 0.528 |
| Age | 0.00029 | 3.4e-05 | 0.78 | 2.2e-11 | 0.00028 | 3.4e-05 | 0.76 | 4.72e-11 |
| Sex | 1.8e-05 | 0.00023 | 0.0071 | 0.938 | 8.7e-05 | 0.00023 | 0.034 | 0.711 |
| Ind. ASD |  |  |  |  | -2.7e-05 | 1.6e-05 | -0.15 | 0.107 |
| R2 | 0.61 |  |  |  | 0.63 |  |  |  |
|  | R2 Change | F | p | adj. p |  |  |  |  |
|  | 0.02 | 2.7 | 0.107 | 0.226 |  |  |  |  |
| **PS** |  | **Model 1** |  |  |  | **Model 2** |  |  |
|  | B | SE | Std.B | p | B | SE | Std.B | p |
| Constant | 320 | 50 |  | 6.59e-08 | 300 | 55 |  | 1.11e-06 |
| Age | 0.46 | 3.2 | 0.021 | 0.887 | 0.7 | 3.3 | 0.032 | 0.832 |
| Sex | 4.8 | 22 | 0.032 | 0.827 | 2.8 | 23 | 0.018 | 0.903 |
| Ind. ASD |  |  |  |  | 0.82 | 1.6 | 0.076 | 0.61 |
| R2 | 0.0011 |  |  |  | 0.0065 |  |  |  |
|  | R2 Change | F | p | adj. p |  |  |  |  |
|  | 0.0053 | 0.26 | 0.610 | 0.757 |  |  |  |  |

| **TPS** |  | **Model 1** |  |  |  | **Model 2** |  |  |
| --- | --- | --- | --- | --- | --- | --- | --- | --- |
|  | B | SE | Std.B | p | B | SE | Std.B | p |
| Constant | 1 | 0.14 |  | 7.39e-09 | 1 | 0.16 |  | 3.18e-08 |
| Age | 0.046 | 0.0093 | 0.59 | 7.72e-06 | 0.046 | 0.0094 | 0.58 | 1.41e-05 |
| Sex | 0.1 | 0.064 | 0.19 | 0.113 | 0.11 | 0.065 | 0.2 | 0.0988 |
| Ind. ASD |  |  |  |  | -0.0027 | 0.0046 | -0.071 | 0.555 |
| R2 | 0.33 |  |  |  | 0.34 |  |  |  |
|  | R2 Change | F | p | adj. p |  |  |  |  |
|  | 0.0048 | 0.35 | 0.555 | 0.740 |  |  |  |  |
| **RT** |  | **Model 1** |  |  |  | **Model 2** |  |  |
|  | B | SE | Std.B | p | B | SE | Std.B | p |
| Constant | 1.4 | 0.2 |  | 7.1e-09 | 1.5 | 0.22 |  | 1.95e-08 |
| Age | 0.095 | 0.013 | 0.73 | 2.88e-09 | 0.093 | 0.013 | 0.72 | 6.8e-09 |
| Sex | 0.21 | 0.09 | 0.23 | 0.0252 | 0.22 | 0.092 | 0.25 | 0.0195 |
| Ind. ASD |  |  |  |  | -0.0055 | 0.0065 | -0.087 | 0.399 |
| R2 | 0.51 |  |  |  | 0.52 |  |  |  |
|  | R2 Change | F | p | adj. p |  |  |  |  |
|  | 0.0071 | 0.72 | 0.399 | 0.624 |  |  |  |  |
| **PA** |  | **Model 1** |  |  |  | **Model 2** |  |  |
|  | B | SE | Std.B | p | B | SE | Std.B | p |
| Constant | 0.31 | 0.11 |  | 0.00574 | 0.33 | 0.12 |  | 0.0077 |
| Age | 0.034 | 0.0069 | 0.57 | 1.02e-05 | 0.033 | 0.007 | 0.57 | 1.74e-05 |
| Sex | -0.033 | 0.047 | -0.08 | 0.495 | -0.029 | 0.049 | -0.072 | 0.55 |
| Ind. ASD |  |  |  |  | -0.0013 | 0.0034 | -0.046 | 0.698 |
| R2 | 0.36 |  |  |  | 0.36 |  |  |  |
|  | R2 Change | F | p | adj. p |  |  |  |  |
|  | 0.002 | 0.15 | 0.698 | 0.771 |  |  |  |  |

| **Mean TE** |  | **Model 1** |  |  |  | **Model 2** |  |  |
| --- | --- | --- | --- | --- | --- | --- | --- | --- |
|  | B | SE | Std.B | p | B | SE | Std.B | p |
| Constant | 0.053 | 0.026 |  | 0.0446 | 0.061 | 0.027 |  | 0.025 |
| Age | 0.007 | 0.0017 | 0.51 | 0.000124 | 0.0068 | 0.0017 | 0.49 | 0.000187 |
| Sex | -0.005 | 0.012 | -0.052 | 0.67 | -0.0058 | 0.011 | -0.061 | 0.618 |
| Anxiety Count |  |  |  |  | -0.0011 | 0.00089 | -0.15 | 0.217 |
| R2 | 0.27 |  |  |  | 0.29 |  |  |  |
|  | R2 Change | F | p | adj. p |  |  |  |  |
|  | 0.022 | 1.6 | 0.217 | 0.372 |  |  |  |  |
| **IIV of TE** |  | **Model 1** |  |  |  | **Model 2** |  |  |
|  | B | SE | Std.B | p | B | SE | Std.B | p |
| Constant | 0.044 | 0.056 |  | 0.44 | 0.053 | 0.058 |  | 0.367 |
| Age | 0.013 | 0.0036 | 0.44 | 0.00109 | 0.012 | 0.0037 | 0.43 | 0.00149 |
| Sex | -0.0055 | 0.025 | -0.028 | 0.827 | -0.0064 | 0.025 | -0.033 | 0.8 |
| Anxiety Count |  |  |  |  | -0.0013 | 0.002 | -0.083 | 0.515 |
| R2 | 0.2 |  |  |  | 0.21 |  |  |  |
|  | R2 Change | F | p | adj. p |  |  |  |  |
|  | 0.0068 | 0.43 | 0.515 | 0.713 |  |  |  |  |
| **NJ** |  | **Model 1** |  |  |  | **Model 2** |  |  |
|  | B | SE | Std.B | p | B | SE | Std.B | p |
| Constant | 0.00027 | 0.00054 |  | 0.617 | 0.00045 | 0.00055 |  | 0.422 |
| Age | 0.00028 | 3.5e-05 | 0.75 | 1.94e-10 | 0.00027 | 3.5e-05 | 0.73 | 3.3e-10 |
| Sex | -8.7e-05 | 0.00024 | -0.034 | 0.719 | -1e-04 | 0.00024 | -0.041 | 0.663 |
| Anxiety Count |  |  |  |  | -2.4e-05 | 1.9e-05 | -0.12 | 0.194 |
| R2 | 0.57 |  |  |  | 0.58 |  |  |  |
|  | R2 Change | F | p | adj. p |  |  |  |  |
|  | 0.014 | 1.7 | 0.194 | 0.866 |  |  |  |  |
| **PS** |  | **Model 1** |  |  |  | **Model 2** |  |  |
|  | B | SE | Std.B | p | B | SE | Std.B | p |
| Constant | 310 | 48 |  | 3.51e-08 | 300 | 50 |  | 1.56e-07 |
| Age | 0.62 | 3.1 | 0.028 | 0.845 | 0.84 | 3.2 | 0.038 | 0.792 |
| Sex | 6.1 | 21 | 0.04 | 0.779 | 6.9 | 22 | 0.046 | 0.75 |
| Anxiety Count |  |  |  |  | 1.2 | 1.7 | 0.1 | 0.485 |
| R2 | 0.0019 |  |  |  | 0.012 |  |  |  |
|  | R2 Change | F | p | adj. p |  |  |  |  |
|  | 0.0098 | 0.5 | 0.485 | 0.698 |  |  |  |  |

| **TPS** |  | **Model 1** |  |  |  | **Model 2** |  |  |
| --- | --- | --- | --- | --- | --- | --- | --- | --- |
|  | B | SE | Std.B | p | B | SE | Std.B | p |
| Constant | 1 | 0.14 |  | 1.43e-09 | 1.1 | 0.15 |  | 3.71e-09 |
| Age | 0.044 | 0.0092 | 0.57 | 1.45e-05 | 0.044 | 0.0094 | 0.57 | 2.03e-05 |
| Sex | 0.087 | 0.063 | 0.16 | 0.175 | 0.086 | 0.064 | 0.16 | 0.183 |
| Anxiety Count |  |  |  |  | -0.001 | 0.005 | -0.025 | 0.834 |
| R2 | 0.31 |  |  |  | 0.31 |  |  |  |
|  | R2 Change | F | p | adj. p |  |  |  |  |
|  | 0.00061 | 0.044 | 0.834 | 0.367 |  |  |  |  |
| **RT** |  | **Model 1** |  |  |  | **Model 2** |  |  |
|  | B | SE | Std.B | p | B | SE | Std.B | p |
| Constant | 1.5 | 0.21 |  | 1.45e-09 | 1.6 | 0.21 |  | 1.03e-09 |
| Age | 0.09 | 0.013 | 0.7 | 1.56e-08 | 0.088 | 0.013 | 0.69 | 2.62e-08 |
| Sex | 0.17 | 0.092 | 0.2 | 0.0665 | 0.17 | 0.092 | 0.19 | 0.076 |
| Anxiety Count |  |  |  |  | -0.0089 | 0.0071 | -0.13 | 0.216 |
| R2 | 0.47 |  |  |  | 0.49 |  |  |  |
|  | R2 Change | F | p | adj. p |  |  |  |  |
|  | 0.016 | 1.6 | 0.216 | 0.372 |  |  |  |  |
| **PA** |  | **Model 1** |  |  |  | **Model 2** |  |  |
|  | B | SE | Std.B | p | B | SE | Std.B | p |
| Constant | 0.35 | 0.11 |  | 0.0018 | 0.4 | 0.11 |  | 0.000447 |
| Age | 0.032 | 0.0069 | 0.54 | 2.97e-05 | 0.03 | 0.0068 | 0.52 | 4.14e-05 |
| Sex | -0.048 | 0.047 | -0.12 | 0.315 | -0.053 | 0.046 | -0.13 | 0.255 |
| Anxiety Count |  |  |  |  | -0.0071 | 0.0036 | -0.22 | 0.0553 |
| R2 | 0.33 |  |  |  | 0.38 |  |  |  |
|  | R2 Change | F | p | adj. p |  |  |  |  |
|  | 0.048 | 3.9 | 0.055 | 0.142 |  |  |  |  |

TE: Tracking Error, IIV of TE: Intra-Individual Variability of Tracking Error, NJ: Normalised Jerk, TPS: Time to Peak Speed, RT: Reaction Time, PA: Path Accuracy
